# Improved multi-trait prediction of wheat end-product quality traits by integrating NIR-predicted phenotypes

**DOI:** 10.3389/fpls.2023.1167221

**Published:** 2023-05-18

**Authors:** Shiva Azizinia, Daniel Mullan, Allan Rattey, Jayfred Godoy, Hannah Robinson, David Moody, Kerrie Forrest, Gabriel Keeble-Gagnere, Matthew J. Hayden, Josquin FG. Tibbits, Hans D. Daetwyler

**Affiliations:** ^1^ Agriculture Victoria, AgriBio, Centre for AgriBioscience, Bundoora, VIC, Australia; ^2^ InterGrain, Bibra Lake, WA, Australia; ^3^ School of Applied Systems Biology, La Trobe University, Bundoora, VIC, Australia

**Keywords:** genomic prediction, multi-trait model, wheat breeding, genomic best linear unbiased prediction, NIR-predictor, forward-prediction, end-product quality traits

## Abstract

Historically, end-product quality testing has been costly and required large flour samples; therefore, it was generally implemented in the late phases of variety development, imposing a huge cost on the breeding effort and effectiveness. High genetic correlations of end-product quality traits with higher throughput and nondestructive testing technologies, such as near-infrared (NIR), could enable early-stage testing and effective selection of these highly valuable traits in a multi-trait genomic prediction model. We studied the impact on prediction accuracy in genomic best linear unbiased prediction (GBLUP) of adding NIR-predicted secondary traits for six end-product quality traits (crumb yellowness, water absorption, texture hardness, flour yield, grain protein, flour swelling volume). Bread wheat lines (1,400–1,900) were measured across 8 years (2012–2019) for six end-product quality traits with standard laboratory assays and with NIR, which were combined to generate predicted data for approximately 27,000 lines. All lines were genotyped with the Infinium™ Wheat Barley 40K BeadChip and imputed using exome sequence data. End-product and NIR phenotypes were genetically correlated (0.5–0.83, except for flour swelling volume 0.19). Prediction accuracies of end-product traits ranged between 0.28 and 0.64 and increased by 30% through the inclusion of NIR-predicted data compared to single-trait analysis. There was a high correlation between the multi-trait prediction accuracy and genetic correlations between end-product and NIR-predicted data (0.69–0.77). Our forward prediction validation revealed a gradual increase in prediction accuracy when adding more years to the multi-trait model. Overall, we achieved genomic prediction accuracy at a level that enables selection for end-product quality traits early in the breeding cycle.

## Highlights

- Including NIR-predicted data into multi-trait prediction models increases the prediction accuracy of genetically correlated end-product quality traits in wheat, supporting the selection of desirable lines in early breeding cycles

## 1 Introduction

An exponentially growing human population and a rapidly changing and more variable climate present major risks to food security. Wheat is the most important grain food source for humans and is used for a diversity of products (FAO, 2018). World wheat production will need to increase by 60% by 2050 to feed over 9.5 billion people (Gerland et al., 2014), which has to be achieved in an increasingly variable environment with more extreme weather conditions and land scarcity (Ceccarelli et al., 2010; Guo et al., 2020).

Current major objectives in wheat breeding include enhanced grain yield, improved agronomic performance, and durable disease resistance, all production-oriented traits. While of considerable value, breeding for end-product quality traits is generally a secondary target met through the application of quality thresholds for release and consumer acceptance (Battenfield et al., 2016). As such, ensuring the breeding focus is from a whole value chain perspective through the incorporation of end-product quality traits into early-generation selection will have a major impact on value creation through breeding. A major obstacle in measuring end-product quality traits, such as flour quality characteristics, is that they often involve time-consuming, labor-intensive, and costly assays that require large grain samples. Thus, sophisticated quality tests have to be postponed to the later phases of variety development. It is common that candidate wheat lines, for which considerable investment in testing has been made, do not pass quality thresholds and are therefore not released to growers. Overcoming these significant limitations requires tools to enable discrimination against undesirable lines earlier in the breeding cycle, improve these traits, and save both time and resources.

In complex genetic phenotypes, the application of marker-assisted selection (MAS) is limited due to its low power to detect minor quantitative trait loci (QTL), genotype-by-environment interactions, and genotype-by-genotype interaction (interaction of QTL and plant genetic background). Numerous linkage mapping studies have shown that a large number of QTL with small effects control most end-product quality traits, and, therefore, MAS is unlikely to be able to capture sufficient variance of these traits to be useful in breeding programs (Carter et al., 2012; Jernigan et al., 2018; Kristensen, 2018; Yang et al., 2020). Genomic selection (GS) can now be routinely implemented through the establishment of prediction models based on a suitable training population with both phenotypic and genotypic data. The trained model is used to predict genomic estimated breeding values (GEBVs) of individuals with only genotypic information (Meuwissen et al., 2001). The effectiveness of GS in complex trait breeding, in accelerating breeding cycles, and in improving genetic gains per unit of time has been proven (Battenfield et al., 2016; Cericola et al., 2017; Michel et al., 2017; Belamkar et al., 2018). The use of GS is particularly advantageous in early generations, which could support breeders in the faster development of new bread wheat varieties that efficiently combine superior baking quality with higher grain yield.

When compared to single-trait analysis, multivariate and/or multi-environment predictive models generally have improved accuracies, especially for traits with high genetic correlations (Guo et al., 2014; Hayes et al., 2017; Okeke et al., 2017; Azizinia et al., 2020; Guo et al., 2020). High genetic correlations are common for quantitative traits such as grain yield and nutritional content in cereal crops (Jia and Jannink, 2012; Lozada and Carter, 2019; Guo et al., 2020). The advantage of multi-variate models could be further extended where the selection population was already phenotyped for a correlated trait (Jia and Jannink, 2012; Hayes et al., 2017). The use of correlated traits is especially useful for predicting expensive or difficult-to-measure traits (Lado et al., 2018; Lozada and Carter, 2019), such as many quality traits (Battenfield et al., 2016; Bhatta et al., 2020). Predictors from near-infrared resonance (NIR) can be treated as a correlated trait in multi-trait models (Dowell et al., 2006; Osborne, 2006; Hayes et al., 2017) and can be easily deployed at scale as they are higher throughput, nondestructive, and require only a small quantity of whole grain when compared to end-use quality assays. Many empirical studies have shown that increasing the size of the training population and/or improving the relationship between the training and prediction populations has a positive impact on prediction accuracy (Habier et al., 2007; Heffner et al., 2011; Clark et al., 2012; Lorenz et al., 2012; Hoffstetter et al., 2016). Here, we investigate the impact on the prediction accuracy of wheat quality traits by adding correlated NIR-predicted data to increase the training population size. This increase in size may also be improving the relationship between the training and prediction sets; however, these effects have not been separated in our analysis.

Cross-validation schemes can be implemented that mimic real breeder circumstances when predicting lines in environments/years that have not been observed in the field. In forward prediction, previous years are used to predict the following year’s progeny. This method represents a common breeding situation where previous data are available, but no data are available for phenotypes or environments in the future year(s). Predicting the performance of lines for future years is a significant missing data problem and is challenging (Hoffstetter et al., 2016; Jarquín et al., 2017; Juliana et al., 2018). Here, we explored the potential of genomic selection to predict future performance using data from 2012 to 2019 for end-product quality traits in fivefold cross-validation and forward prediction.

The main objectives of this study were to (1) estimate the genetic correlations of end-product quality and NIR-predicted data, (2) evaluate the influence of adding NIR-predicted data in improving prediction accuracies of end-product quality traits using fivefold cross-validation, and (3) investigate the forward prediction of line performance across years in a multi-environment context.

## 2 Material and methods

### 2.1 Plant materials and phenotyping

Phenotype records for six quality traits from hexaploid bread wheat breeding lines measured in laboratory assays along with NIR-predicted data from approximately 27,000 lines were used in this study. Wheat lines were evaluated for 8 years from 2012 to 2019, grown in NSW, VIC, WA, and SA, in more than 150–197 trials of end-product traits and 409–450 trials for NIR-predicted data. Lines were evaluated for crumb yellow/blueness (b*; color), flour water absorption (Wab; %), hardness/particle size index of flour (PSI; %), flour yield (FlrYld; %), protein (Protein; %), and flour swelling volume (FSV, ml/g). All data across trials and years were used in the analysis, with a summary of the number of records and lines in each trait and their range and means provided in **Tables 1**, **2**.

**Table 1:** Number of lines used for genomic analysis.

Traits	Years	b*	Wab	PSI	FlrYld	Protein	FSV
End-product	2012	29	29	29	28	18	29
	2013	188	189	189	189	189	188
	2014	169	169	169	169	169	166
	2015	219	223	177	251	219	147
	2016	161	161	194	209	156	155
	2017	166	164	304	309	114	131
	2018	308	308	233	379	308	302
	2019	401	352	469	400	407	284
**Sum**		**1,641**	**1,595**	**1,764**	**1,934**	**1,580**	**1,402**
NIR predicted	2012	29	29	29	29	29	16
	2013	80	80	80	80	80	5
	2014	92	86	86	86	86	84
	2015	114	114	114	114	114	114
	2016	249	249	249	249	249	249
	2017	439	439	439	439	439	439
	2018	8375	8375	8375	8374	8375	8358
	2019	17,653	17,653	17,651	17,653	17,653	17,653
**Sum**		**27,031**	**27,025**	**27,023**	**27,024**	**27,025**	**26,918**

b*, crumb yellow/blueness; Wab, water absorption; PSI, texture hardness (particle size index); FlrYld, flour yield; Protein, grain protein; FSV, flour swelling volume.

**Table 2:** End-product quality trait ranges (Min, minimum; Max, mean and maximum); variance components (genetic variance: $\sigma_{g}^{2}$ and residuals: $\sigma_{e}^{2}$ ); and broad- (*H* ^2^) and narrow-sense heritability (*h* ^2^) estimated with the SNP of lines analyzed from 2012 to 2019.

Trait	Min	Mean	Max	$\sigma_{g}^{2}$	$\sigma_{e}^{2}$	*H* ^2^	*h* ^2^ (SNP)
b* (colo)	5.96	9.62	18.70	0.86	0.13	0.87	0.68
**Wab** (%)	48.90	60.74	71.10	1.9	0.91	0.68	0.67
**PSI (**%)	7.00	15.68	36.80	3.64	1.47	0.71	0.68
**FlrYld (**%)	65.03	74.14	80.61	0.93	0.51	0.65	0.54
**Protein** (%)	7.55	11.47	15.80	0.19	0.30	0.40	0.40
**FSV (**ml/g)	7.14	12.31	29.00	8.42	8.31	0.50	0.27

Laboratory assays were conducted on 2–4 kg composite samples assembled by blending all replicated plots of each line per trial. The samples were conditioned at 16% moisture content for 24 h prior to milling using a Buhler Laboratory Mill (MLU 202). End-use quality tests were performed using approved methods of the American Association of Cereal Chemists International (AACC International, 2008). The PSI (%), a measure of wheat hardness, was determined after grinding and sieving of grain samples (AACC Method 55-30.01). FlrYld (%) is the percentage by weight recovered of the total product as straight-grade white flour. b* was analyzed with a Minolta Chroma Meter (C-100, Minolta Camera Co. Ltd., Osaka, Japan) (Oliver et al., 1992). FSV (ml/g) test was performed using AACC Method 56-21.01, and Wab (%) was measured using a Farinograph (Brabender, Germany) following AACC Method 54-21.02. Near-infrared spectroscopy data were also acquired for each of the end-use quality traits. NIR predictions were generated by loading 100 grams of sample into the XDS Rapid Content Analyzer (FOSS). AACC Methods 39-25.01 and 39-70.02 were used to determine NIR-predicted protein content and PSI of whole grains using a local calibration.

### 2.2 Genotyping

Genomic DNA was extracted from six seeds per sample using a modified CTAB method (Tibbits et al., 2006), where a magnetic bead clean-up step replaced isopropanol precipitation. In brief, the modifications consisted of mixing 120 µl of the upper aqueous phase with 120 µl of 10× diluted AMPure XP beads (Beckman Coulter Inc. CA, USA) (diluted with a solution containing 20% PEG and 2.5 M NaCl), followed by one wash with 200 µl of 50% DNAzol^®^ ES (Molecular Research Centre Inc. OH, USA) and 42.5% ethanol and two washes with 70% ethanol. DNA was eluted in 15 µl of 10 mM Tris-HCl at pH 8.0.

The reference panel was genotyped with the Illumina Infinium Wheat Barley 40K XT SNP array v1.0 (Keeble-Gagnère et al., 2021) according to the manufacturer’s instructions (Illumina Ltd., CA, United States), with modifications detailed in Keeble-Gagnère et al. (2021). Genotype calling was performed using a custom pipeline (Maccaferri et al., 2019; Keeble-Gagnère et al., 2021).

Genotypes were imputed to exome density following the procedure described in Keeble-Gagnère et al. (2021). Briefly, sporadic missing data were filled in with Beagle v4.1 (Browning and Browning, 2016), before converting SNP coordinates to positions in the IWGSC RefSeq v2.0 (O’Leary et al., 2016) assembly and imputing to 435,404 SNPs with Minimac 3 (Das et al., 2016) using the reference haplotypes described in Keeble-Gagnère et al. (2021) together with 102 exome-sequenced historical lines from InterGrain’s breeding program. This target set of 435,404 SNPs was defined as the set of SNPs with $r^{2}>0.7$ to the set of genotyped SNPs, based on the LD in the InterGrain historical lines. This set of SNPs was further reduced to 330,169 after selecting the SNPs in common with the transcriptome genotyping-by-sequencing (tGBS) genotypes after imputation.

### 2.3 Broad-sense heritability

Phenotypic records were edited for possible outliers (mean ± 4SD). Phenotypes were also adjusted for fixed effects using a linear mixed model (Eq. 1) in ASReml (Butler et al., 2017), where **y** was a vector of quality phenotypes, *µ* was the trait mean, trial was a group effect of year, location, and nursery and fitted as fixed effect in the model.


(1) $$y=\mu +Trials_{\left(year+location+nursery\right)}+line+e$$

The line was fitted as fixed to estimate BLUEs as adjusted phenotypes in GEBV estimation and random to estimate the variance due to lines in order to determine the broad sense heritability using:


(2) H2=σg2σg2+σg2TR

where $\sigma_{g}^{2}$ and $\sigma_{e}^{2}$ are line and residual variances, respectively. T and R are the mean numbers of trials and replications per line.

### 2.4 Genomic predictions

Genomic Restricted Maximum Likelihood (GREML) was used for estimating GEBVs and variance components in the MTG2 software (Lee and van der Werf, 2016).

### 2.5 Single-trait genomic prediction

The single-trait GREML model used in this study was a linear mixed model described as


(3) $$y=1_{n}\mu +Zu+e,$$

where y is the vector of adjusted BLUEs for the trait, *μ* is the overall mean, **1** *_n_* is a vector of ones, **Z** is a design matrix relating records to breeding values, **u** is a vector of GEBVs, and **e** is a vector of residual effects. It was assumed that **u**~ ***N*** (**0**, $G\sigma_{g}^{2}$ ), where $\sigma_{g}^{2}$ is additive genetic variance and **G** is the genomic relationship matrix calculated as described in Yang et al. (2010) from the 330,169 SNP markers, and **e**~N (**0**, $I\sigma_{e}^{2}$ ), where $\sigma_{e}^{2}$ is the residual variance and **I** is the *n* × *n* identity matrix. We performed a principal component analysis (PCA) of **G** using the prcomp function in **R** (R Core Team, 2020) to investigate the population structure in the sample. A plot of the first two components was visualized using the ggplot2 package (Wickham, 2016) in R. Narrow-sense heritability (*h* ^2^) was calculated as the ratio of the additive variance to the total phenotypic variance using the GREML model.

**Figure 1:**
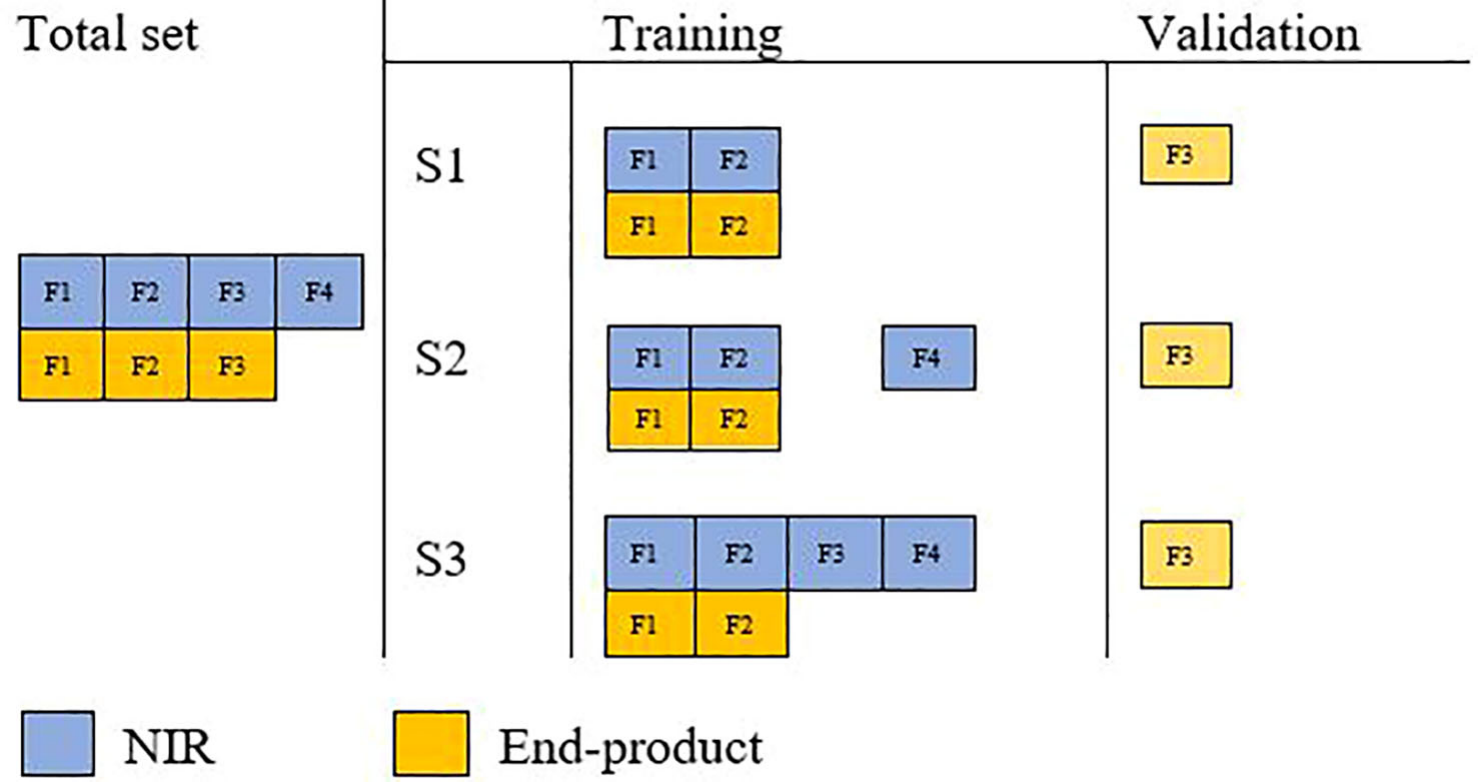
Schematic illustration of cross-validation in three different multi-trait analysis scenarios.

Single-trait genomic prediction accuracy was evaluated with fivefold random cross-validation. In each cross-validation run, onefold was used as a validation set with their phenotype data masked in the analysis (**Figure 1**). The accuracy of genomic prediction was calculated as the Pearson correlation coefficient between corrected phenotypic values and GEBVs in the validation subset. This process was repeated 10 times and average prediction accuracy across all folds (50) was reported.

### 2.6 Multi-trait genomic prediction

A basic multi-trait mixed model was used as follows:


(4) $$\left[\begin{array}{c} y_{\text{E}P}\\ y_{\text{NIR}} \end{array}\right]=\left[\begin{array}{cc} 1_{\text{EP}} & 0\\ 0 & 1_{\text{NIR}} \end{array}\right]\left[\begin{array}{c} {\text{μ}}_{\text{EP}}\\ {\text{μ}}_{\text{NIR}} \end{array}\right]+\left[\begin{array}{cc} Z_{\text{EP}} & 0\\ 0 & Z_{\text{NIR}} \end{array}\right]\left[\begin{array}{c} u_{\text{EP}}\\ u_{\text{NIR}} \end{array}\right]+\left[\begin{array}{c} e_{\text{EP}}\\ e_{\text{NIR}} \end{array}\right]$$

where **y**_EP_ and **y**_NIR_ are the vectors of adjusted phenotypes, **1** is a vector of ones, *μ*_EP_ and *μ*_NIR_ are general means, **Z**_EP_ and **Z**_NIR_ are the design matrices of breeding values, **u**_EP_ and **u**_NIR_ are the vectors of genomic breeding values, and **e**_EP_ and **e**_NIR_ are the vectors of random residual effects, for trait end-product quality and NIR-predicted data, respectively. Residuals ( $e=[e_{EP},e_{NIR}]$ ) are assumed to follow a normal distribution, **e** | **R**_0_ ~ N(**0**, R_0_ ⊗ **I**) and


$$R_{0}=[\begin{array}{cc} \sigma_{e_{EP}}^{2} & \sigma_{e_{EP-NIR}}^{2}\\ \sigma_{e_{NIR-EP}}^{2} & \sigma_{e_{NIR}}^{2} \end{array}].$$

Residuals are assumed to be unstructured.

Wheat end-product quality traits are costly to assay directly and therefore cannot be rapidly determined for a large number of lines in breeding programs. NIR-predicted data can be generated easily across many lines and used to predict end-use traits, making them an interesting test case for examining bivariate models. The benefit of using NIR to predict end-product quality traits was investigated using three different cross-validation scenarios.

#### 2.6.1 Cross-validation scenario S1

The end-product quality phenotypes of validation lines were masked and predicted with a reference population with equal numbers of end-product and NIR-predicted data. This aimed to assess the effect of multi-trait analysis in the improvement of prediction accuracy of end-product quality traits where both traits are available on the same lines (i.e., their assessment is costly and time-consuming).

#### 2.6.2 Cross-validation scenario S2

Validation lines were masked from both end-product and NIR-predicted traits; however, the reference set included all additional lines with only NIR-predicted data. This aimed to study the effect of extra NIR-predicted data on improving prediction accuracy.

#### 2.6.3 Cross-validation scenario S3

Assessment of adding NIR-predicted data on validation lines to increase prediction accuracy, assuming that NIR-predicted data are easy and cost-effective to generate on all lines. In this scenario, end-product phenotypes were masked only in the validation set, while their corresponding NIR measurements were included in the reference set.

### 2.7 Forward genomic prediction

In breeding programs, the aim is to predict future years/environments using previous years’ data. These independent predictions were performed by training the model on previous years’ data and predicting future environments using GREML. Data from 2012 to 2015 were used to predict 2016, 2012 to 2016 to predict 2017, and 2012 to 2018 to predict 2019.

## 3 Results

### 3.1 Trait heritability and correlation

Phenotype records of wheat lines from different breeding cycles (2012–2019) were used to estimate broad- and narrow-sense heritabilities as well as genomic prediction analyses. Records were unbalanced across years and trials, with, in total, 1,400–1,900 end-product wheat lines and 27,000 NIR-predicted lines available. For both end-product and NIR-predicted data, the lowest number of individuals was recorded in 2012, while in 2018 and 2019, the largest number of lines were measured. Phenotypic records by trait and year are summarized in **Table 1**. Phenotypic values exhibited variation suitable for breeding, with *H* ^2^ being high for b*, Wab, and PSI, moderate for FlrYld and Protein, and relatively low for FSV (**Table 2**). SNPs also captured a high proportion of the phenotypic variance, as demonstrated by *h* ^2^.

Genetic correlations between end-product quality and NIR-predicted traits were estimated using a multi-trait model (**Table 3**). All end-product traits were strongly correlated with NIR-predicted traits, with the exception of FSV, which had a low correlation, and protein, with a moderate correlation. The phenotypic correlations for the trait sets were high for most traits, except for FlrYld and FSV (**Table 3**).

**Table 3:** Phenotypic and genetic correlations between end-product and NIR-predicted traits.

Trait	Genetic correlation	Phenotypic correlation
b*	0.83 ± 0.02	0.46 ± 0.01
Wab	0.86 ± 0.02	0.67 ± 0.01
PSI	0.82 ± 0.03	0.69 ± 0.01
FlrYld	0.67 ± 0.04	0.26 ± 0.01
Protein	0.50 ± 0.06	0.85 ± 0.01
FSV	0.19 ± 0.08	0.12 ± 0.02

### 3.2 Population structure

**Figure 2:**
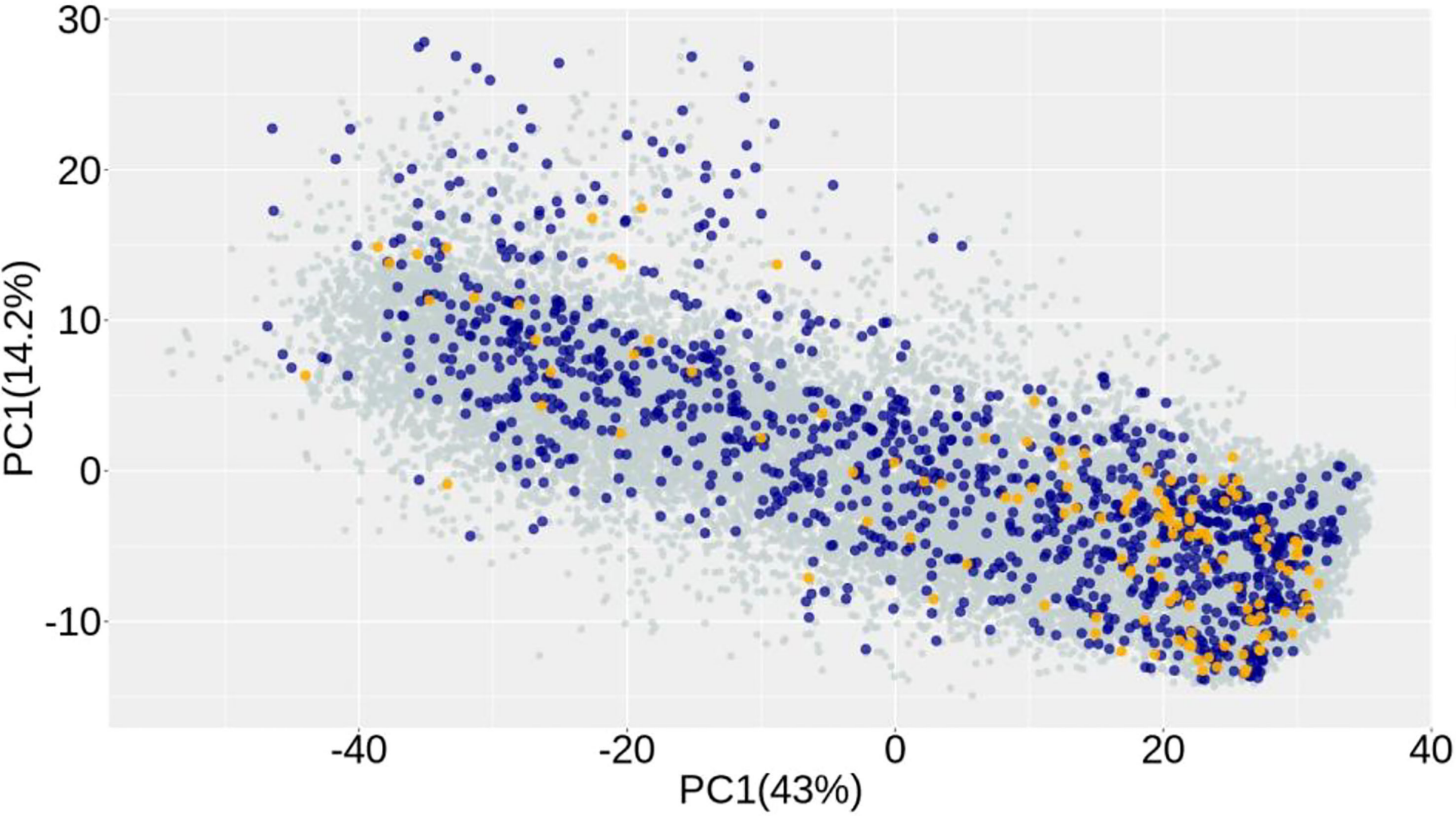
Principal component analysis of the genomic relationship matrix of wheat lines showing the distribution of lines with end-product (yellow), NIR (aqua) phenotypic data, or with both traits (dark blue).

A principal component analysis was used to investigate population structure. The two first components jointly explained 56.2% of the variation (**Figure 2**). The plot shows the relationship between groups of individuals used in the analysis. There is a slightly denser concentration of lines in the lower right corner of the chart for three groups (i.e., end-product only, NIR only, and both phenotypes). However, there is a complete overlap of lines in all groups. In other words, lines from diverse backgrounds have been recorded for both NIR and end-product performance. This close genetic relationship between lines with end-product assay data and those with NIR-predicted data is expected to improve prediction accuracy and result in more accurate genomic breeding values.

### 3.3 Single-trait prediction

Single-trait BLUE prediction accuracies for end-product quality traits were highly variable, ranging from 0.20 for FSV to 0.56 for b* (**Table 4**). A correlation between trait heritability and prediction accuracy was observed with traits of lower heritability tending towards lower prediction accuracy. The correlations of broad- and narrow-sense heritabilities with single-trait prediction accuracies were 0.62 and 0.54, respectively.

**Table 4:** Mean forward prediction accuracies across 2016–2019 in single- and multi-trait analyses.

	b*	Wab	PSI	Flour yield	Protein	FSV
Multi-trait	0.54	0.09	0.42	0.17	0.21	0.16
Single trait	0.49	0.07	0.42	0.17	0.19	0.15

### 3.4 Multi-trait prediction

**Figure 3:**
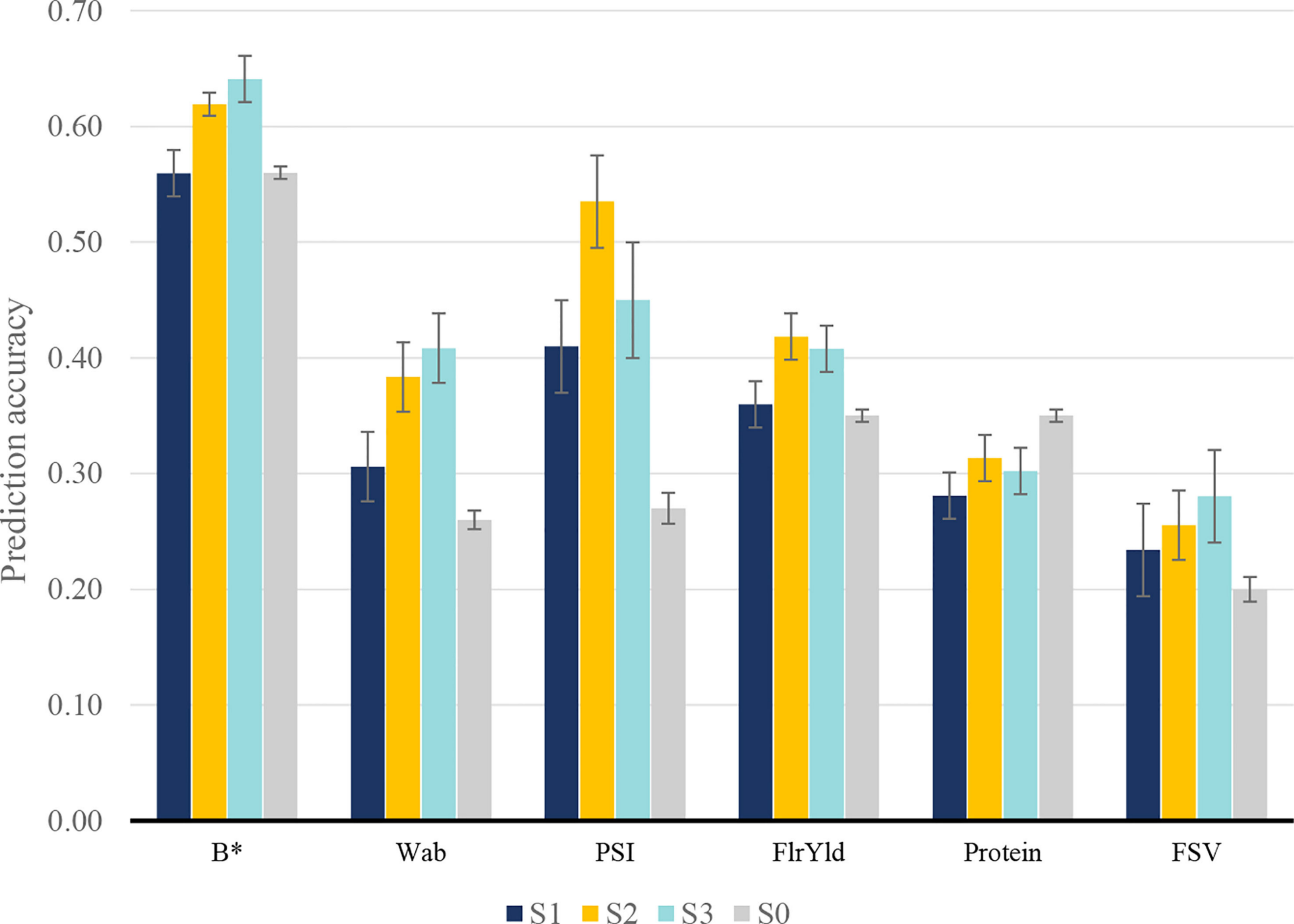
Prediction accuracy for wheat end-product quality traits in S0: single-trait model; S1: using only end-product test results and their corresponding NIR measurement; S2: including extra NIR-predicted data for individuals without end-product test results; and S3: including NIR measurements of the validation set in reference test.

We assessed three scenarios that included NIR-predicted data to predict end-product quality traits in a different manner. In the first scenario (S1), NIR-predicted data were limited to the lines that had been assessed for end-product quality traits, while in the second scenario (S2), a large number of additional lines with NIR-predicted data were added to the training population. In both S1 and S2, end-product measured phenotypes of the validation set and their corresponding NIR-predicted data were removed from training; however, in the third scenario (S3), NIR-predicted data of lines in the validation set were included. The aim of this approach was to investigate whether early NIR phenotyping of candidate lines improves the prediction accuracy of end-product traits. Fivefold cross-validation within the population was used to estimate prediction accuracy from the GBLUP. In S1, adding NIR-predicted data increased the accuracy of predictions in all end-product quality traits compared to single-trait prediction, except for protein, in which there was a reduction in prediction accuracy and b* with no material change (**Figure 3**). In this scenario, PSI showed the highest improvement, followed by Wab.

To identify whether the inclusion of additional NIR-predicted data in the model could further improve the predictive performance of end-product quality traits, S2 and S3 scenarios were studied. Adding more information through the inclusion of NIR-predicted data improved the mean prediction accuracy of all traits (**Figure 3**), although traits responded differently depending on the scenario. For most multi-trait scenarios, the inclusion of NIR-predicted data prediction accuracy increased. Protein content was an exception with multi-trait prediction inferior to the single-trait model in all scenarios. Wab and PSI showed the highest accuracy increase in S2 and S3 when compared to single-trait scenarios, with an average accuracy of 0.40 and 0.50 for Wab and PSI, while their corresponding single-trait accuracy was 0.26 and 0.27, respectively. FlrYld and b* also positively responded to including more NIR-predicted data, where in S3 an accuracy of 0.41 (flour yield) and 0.64 (b*) were observed compared to the single-trait model (0.35 and 0.56, respectively).

The scale of the genetic correlation between end-product and NIR-predicted traits was reflected in the level of genomic prediction accuracy. For example, genetic correlations and prediction accuracies were high for Wab and PSI. There was a high correlation between the magnitude of prediction accuracy in the multi-trait scheme and the genetic correlation of end-product and NIR-predicted data (0.69–0.77). Correlations between broad- and narrow-sense heritability with multi-trait prediction accuracies in different scenarios ranged between 0.53 and 0.60 and 0.43 and 0.47, respectively. Broad- and narrow-sense heritability also had a correlation of 0.58 and 0.45, respectively, with mean prediction accuracies across all three scenarios.

### 3.5 Forward prediction

**Figure 4:**
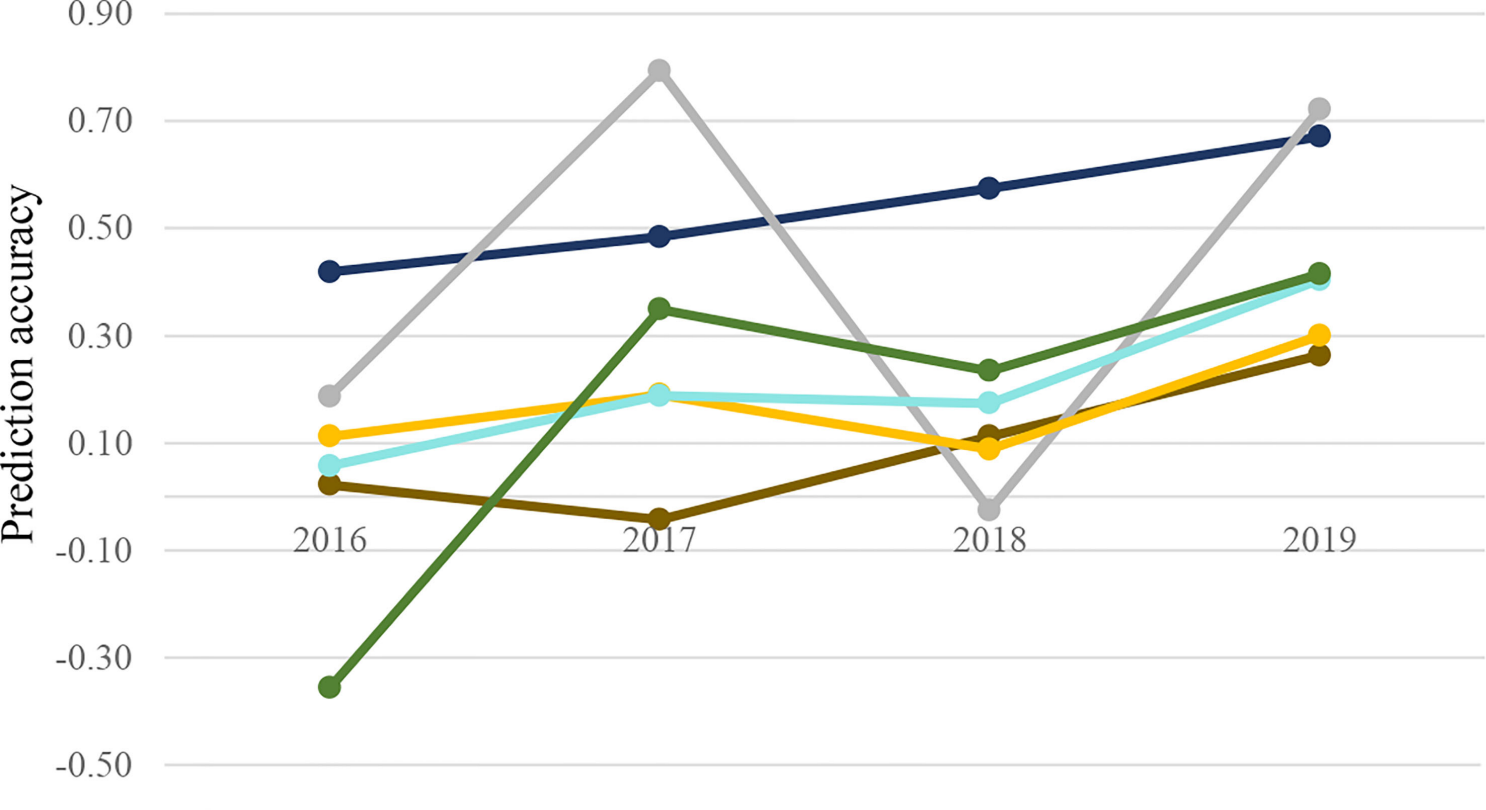
Multi-trait prediction accuracy for wheat end-product quality traits (b*:
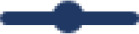
; Wab:
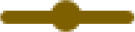
; PSI:
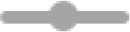
; FlrYld:
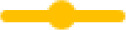
; protein:
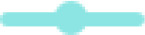
; FSV:
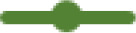
) in forward prediction. Combined data from previous years were applied as training for future years.

Despite some fluctuations, there was a gradual increase in prediction accuracy through the addition of phenotypic data over the years, though PSI accuracy was variable (**Figure 4**). All trait prediction accuracies increased by 13%–30% compared to the mean accuracy across years between 2016 and 2019. The highest forward prediction accuracy was observed for b*, which was similar to single- and multi-trait scenarios.

Forward prediction in a univariate scheme showed similar average prediction accuracy across years with a lower upward trend than the multi-trait, demonstrating more accurate predictions were achieved when using correlated NIR-predicted traits to increase training size (**Table 4**).

## 4 Discussion

Wheat end-product quality traits have been difficult to include in early selection as testing is labor intensive, costly, and requires large grain samples that are usually not available until late in the breeding cycle. These limitations cause breeding program inefficiency with high-yielding lines, which incur significant field trial resources before often being discarded late in the breeding cycle based on quality testing results. The genomic selection offers a new way to include end-use quality traits into the early breeding cycle; however, genomic selection success requires having training populations of sufficient size for accurate prediction, and some of the limitations listed above also make the accumulation of adequate training populations problematic (Hayes et al., 2017; Zhang-Biehn et al., 2021). Studies have also shown that closely related training and validation populations result in accurate genomic breeding values (Habier et al., 2007; Daetwyler et al., 2008; Meuwissen, 2009). In this paper, we demonstrate two effective strategies for increasing the power of genomic selection. First, by increasing the training population size using correlated NIR predictions of end-product quality in a multi-trait model and, second, by adding observations to the training population in a breeding-relevant forward validation scenario across years. Overall, the multi-trait models were effective in increasing prediction accuracy.

### 4.1 Heritability and genetic correlations

The broad-sense heritability of end-product quality traits evaluated varied from 0.40 to 0.87, with most of them having a value above 0.60. These heritability estimates for quality traits are consistent with values reported in other studies (Carter et al., 2012; Jernigan et al., 2018; Michel et al., 2018; Kristensen et al., 2019). The lower heritability of traits like FSV may be representative of a complex and polygenic underlying architecture (Hayes et al., 2017). Intermediate to high heritability estimates suggested that most of the variation of the trait is genetic (Tsai et al., 2020). In this study, the achieved prediction accuracy indicates that genomic selection is suitable for breeding highly heritable traits, as the models are generally able to capture much of the additive genetic variation. Multi-trait models have been shown to improve the prediction performance of traits with lower genetic correlations, whereas correlated predictors have higher heritability (Jia and Jannink, 2012; Guo et al., 2014). However, for very complex polygenic traits, multi-trait models may not show an advantage over single-trait models even where heritability is high (Jia and Jannink, 2012; Lado et al., 2018).

End-product and NIR-predicted traits showed a high genetic correlation. This is consistent with other studies, where the genetic correlation of end-quality traits and their corresponding NIR prediction were notably high (Hayes et al., 2017). High correlations have been shown to have an impact on increasing the prediction accuracy of traits (Hayes et al., 2017; Michel et al., 2018; Azizinia et al., 2020; Guo et al., 2020; Tsai et al., 2020).

In general, traits with lower genetic correlations of end-product and NIR-predicted data showed a smaller improvement in prediction accuracy. This is similar to the result reported by Hayes et al. (2017). Our results also align with previous studies that reported that prediction accuracy for traits with intermediate to low correlations was not substantially improved in multi-trait schemes (Calus and Veerkamp, 2011; Lado et al., 2018). Jia and Jannink (2012) indicated that for very complex traits with low heritability, multi-trait models have little advantage over other models. Our hypothesis was that using a larger reference population would increase prediction power in the validation population with increased relatedness of reference and validation sets. Overall, our results suggest that multi-trait models using correlated attributes do improve the accuracy of genomic prediction (Guo et al., 2014; Rutkoski et al., 2016), and this increases the potential uses of NIR-predicted data to predict wheat end-product quality traits (Dowell et al., 2006; Hayes et al., 2017). Increasing the size of the reference set through the addition of both end-product quality and NIR-predicted data is therefore recommended to improve end-product predictions, especially for traits with moderate and low correlations (e.g., FlrYld, Protein, and FSV).

### 4.2 Multi-trait prediction

Multi-trait models combine information of correlated traits to deliver higher prediction accuracy and are thus useful in enhancing the prediction accuracy of favorable traits, which are difficult to assess in the early cycles of the program. We showed higher prediction accuracy across quality traits in multi-trait models (S1, S2, and S3), which is consistent with Lado et al. (2018), who reported increased prediction ability of quality traits using the information of correlated attributes compared to a single-trait prediction model.

Using NIR-predicted data in a multi-trait scheme, prediction accuracies showed on average a 0.05 to 0.2 (8% to 73%) rise across all scenarios and traits (excluding Protein). This is comparable to Lado et al. (2018), who reported a 60% to 100% increase in prediction accuracy of target quality traits using correlated traits in different multi-trait models. Hayes et al. (2017) also reported an increase of 0.03 to 0.45 (6% to 300%) in soft and hard wheat for grain hardness, grain protein, b*, and water absorption in different multi-trait scenarios, although the strategy used to predict performance was different. Overall, the addition of NIR-predicted data substantially improved trait prediction accuracy, and it is worthwhile perusing as a strategy in breeding programs.

Higher accuracies of different multi-trait scenarios also suggested that an expanded reference set of predictors would improve the prediction accuracy of correlated traits (S2 and S3). While using NIR-predicted data of the reference population (S1) showed an 11% increase in prediction accuracy over the single-trait model, increasing NIR measures as a correlated trait in the second scenario (S2) had a 33% increase over the single model. Including NIR measures of candidate lines in the training population (S3) also showed a 30% increase in prediction accuracy across all traits but did not considerably affect prediction accuracy compared to S2. The additional NIR information in S3 compared to S2 involved adding only a small number of observations. It could be that the addition of a relatively small number of NIR-predicted data would be unlikely to substantially affect the prediction accuracy. Particularly, focusing on improving the genetic correlation of NIR predictions with end-product assays and therefore increasing NIR-predicted data will positively affect prediction accuracy for target traits. The findings of the present study can be potentially applied in plant breeding to achieve more accurate and improved predictions compared to single-trait predictions for end-product quality traits of high importance.

### 4.3 Forward prediction

Predicting the performance of new individuals lacking phenotypes is always a challenge in breeding programs. We demonstrated continuous increases in forward prediction accuracy across years as the size of the training population (and relatedness to the training set) increased. The importance of large training population sizes in increasing forward prediction accuracy has been previously demonstrated (Battenfield et al., 2016; Yao et al., 2018; Michel et al., 2019b). Jarquín et al. (2017) indicated difficulty in predicting future years for grain yield, with lower prediction accuracy compared to cross-validation. In our study, cross-validation in all scenarios had higher prediction accuracy compared to mean forward prediction accuracy. Higher accuracy from cross-validation arises from over-inflation in the method and the fact that the likelihood of assignment of close relatives in validation and reference sets can cause inflation of prediction accuracy (Rutkoski et al., 2015). Lower average forward prediction accuracy compared to cross-validation can be due to the random selection of reference and validation sets in the latter method, which results in a better estimation of environmental variation (Rutkoski et al., 2015; Battenfield et al., 2016). In forward prediction, genotype-by-environment interactions may play a major role since the training set is not as representative of the validation set. On the other hand, the prediction accuracy of 2019 in forward prediction was higher, suggesting the importance of including bigger training sets and genotype–environment interaction, which has been highlighted in other research studies (Crossa et al., 2014). Increasing training population size and updating genomic selection models every year is recommended for the best prediction accuracy (Michel et al., 2019a). Given the promising results of forward prediction for the quality traits, implementing GS will enable breeders to make a selection for those quality traits in a larger population at earlier stages, thereby enhancing selection efficiency (Fiedler et al., 2017). Additionally, eliminating less favorable lines can be done in early cycles of breeding programs based on GEBVs alone. This approach will remove costs from breeding programs by only keeping potential candidate lines that will be of sufficient end-product quality for relevant markets.

Prediction ability could be improved by optimizing other factors affecting GS accuracy (Daetwyler et al., 2008); for example, integrating GxE and spatial effects in the model could improve prediction accuracy (Lin et al., 2021). Extending the multi-trait model to a multi-trait and multi-environment model that takes into account the interaction of trait–genotype–environment is another approach that could enhance the prediction ability of complex traits (Montesinos-lópez et al., 2016; Tsai et al., 2020).

## 5 Conclusion

We investigated the potential benefit of including NIR-predicted data in multi-trait models for predicting six end-product quality traits in a commercial wheat breeding program. Different scenarios of multi-trait models were compared with a single-trait prediction model. We demonstrated that multi-trait models combining direct measures of end-product quality traits with their NIR predictions had higher predictive accuracy than their respective single-trait models. The increased prediction accuracy was observed with the inclusion of NIR-predicted data in the training population, which was likely driven by the increased size of the reference set and the relationship between the reference and validation populations. The prediction accuracies we achieved using the combined data were at a level that breeding selections could be confidently applied in the breeding process to increase breeding efficiency and genetic gain. Integrating NIR-predicted data into the prediction is a cost-effective way to improve the prediction accuracy of end-product quality traits, enabling breeders to confidently validate large numbers of lines in early breeding cycles. While our results state the efficiency of multi-trait analysis in end-quality traits in bread wheat, the method can be generalized to any other plant breeding program that may want to benefit from NIR-predicted data in improving the prediction accuracy of laborious-to-phenotype traits (Hayes et al., 2017; Lado et al., 2018; Azizinia et al., 2020; Sandhu et al., 2022).

## Data availability statement

The data analyzed in this study is subject to the following licenses/restrictions: The datasets analyzed during the current study are not publicly available due to third party commercial restrictions but will be made available from the corresponding author on reasonable request and with permission of Intergrain Pty Ltd. Requests to access these datasets should be directed to shiva.azizinia@agriculture.vic.gov.au.

## Author contributions

SA: analyzed data and wrote the manuscript. DMu, AR, JG, HR, and DMo: provided samples and phenotypes. KF, GK-G, and MH: genotyped samples. MH, JT, and HD: supervised the study. HD: edited the manuscript. All authors have read and approved the manuscript.
