# Supplementary material for: Improved multi-trait prediction of wheat end-product quality traits by integrating NIR-predicted phenotypes
Source: Front Plant Sci. 2023 May 18;14:1167221. doi: 10.3389/fpls.2023.1167221 (PMC10233148; doi:10.3389/fpls.2023.1167221)
Supplement: Supplementary file 1 [file Table_1.docx]

Supplementary information

**TABLE 5 |** prediction accuracies for end-product quality traits with and without using NIR predicted data in reference set.

|  | Multi-trait prediction | | | Single trait prediction |
| --- | --- | --- | --- | --- |
| Trait | S1 | S2 | S3 |  |
| b* | 0.56 | 0.62 | 0.64 | 0.56 |
| Wab | 0.31 | 0.38 | 0.41 | 0.26 |
| PSI | 0.41 | 0.54 | 0.45 | 0.27 |
| FlrYld | 0.36 | 0.42 | 0.41 | 0.35 |
| Protein | 0.28 | 0.31 | 0.30 | 0.35 |
| FSV | 0.23 | 0.26 | 0.28 | 0.20 |
